# Voltage vs. Ligand II: Structural insights of the intrinsic flexibility in cyclic nucleotide-gated channels

**DOI:** 10.1080/19336950.2019.1666456

**Published:** 2019-09-25

**Authors:** Sergio Romero-Romero, Gustavo Martínez-Delgado, Daniel Balleza

**Affiliations:** a Facultad de Medicina, Departamento de Bioquímica, Universidad Nacional Autónoma de México, 04510 Mexico City, Mexico. Current address: Department of Biochemistry, University of Bayreuth, Bayreuth, Germany; b Laboratorio de Genómica de Enfermedades Cardiovasculares, Instituto Nacional de Medicina Genómica, Mexico City, Mexico; c Departamento de Química ICET, Universidad Autónoma de Guadalajara, Zapopan, Jalisco, Mexico

**Keywords:** CNG channels, Local flexibility, voltage sensor, pore domain; *Aquifex*; TolQ

## Abstract

In the preceding article, we present a flexibility analysis of the voltage-gated ion channel (VGIC) superfamily. In this study, we describe in detail the flexibility profile of the voltage-sensor domain (VSD) and the pore domain (PD) concerning the evolution of 6TM ion channels. In particular, we highlight the role of flexibility in the emergence of CNG channels and describe a significant level of sequence similarity between the archetypical VSD and the TolQ proteins. A highly flexible S4-like segment exhibiting Lys instead Arg for these membrane proteins is reported. Sequence analysis indicates that, in addition to this S4-like segment, TolQ proteins also show similarity with specific motifs in S2 and S3 from typical V-sensors. Notably, S3 flexibility profiles from typical VSDs and S3-like in TolQ proteins are also similar. Interestingly, TolQ from early divergent prokaryotes are comparatively more flexible than those in modern counterparts or true V-sensors. Regarding the PD, we also found that 2TM K^+^-channels in early prokaryotes are considerably more flexible than the ones in modern microbes, and such flexibility is comparable to the one present in CNG channels. Voltage dependence is mainly exhibited in prokaryotic CNG channels whose VSD is rigid whereas the eukaryotic CNG channels are considerably more flexible and poorly V-dependent. The implication of the flexibility present in CNG channels, their sensitivity to cyclic nucleotides and the cation selectivity are discussed. Finally, we generated a structural model of the putative cyclic nucleotide-modulated ion channel, which we coined here as AqK, from the thermophilic bacteria *Aquifex aeolicus*, one of the earliest diverging prokaryotes known. Overall, our analysis suggests that V-sensors in CNG-like channels were essentially rigid in early prokaryotes but raises the possibility that this module was probably part of a very flexible stator protein of the bacterial flagellum motor complex.

## Introduction

The voltage-gated ion channel (VGIC) superfamily includes many members containing six-transmembrane (6TM) segments in which the first four form a voltage-sensing domain (VSD) and the last two form the pore domain (PD). This superfamily also includes structurally related channels that are not strictly activated by voltage but instead they are mainly activated by diverse ligands as well as physical stimuli such as temperature and membrane mechanics. Besides, four-domain (DI-DIV) voltage-gated cation channels represent a large family of ion channels where voltage-gated calcium (Ca_v_) and sodium (Na_v_) channels are importantly implicated in electrical excitability, mainly in eukaryotes [1]. The appearance of the first 6TM voltage-gated ion channel has been attributed to the fusion of two distinct modules: (1) a 4TM voltage-gating module and (2) a 2TM pore module. This because both modules also exist as independent functional units, for example, the Hv1 proton channels, the voltage-gated phosphatases, and the inward-rectifying K**^+^** channels [2,3]. In voltage-dependent channels, the fourth transmembrane segment (S4) into the VSD contains several positively-charged residues (*i.e*. Lys or mainly Arg) which sense and respond by gating to changes in the membrane potential [4]. In marked contrast, cyclic nucleotide-gated (CNG) channels are virtually voltage-independent, although they exhibit the typical arrangement of positively charged residues at the S4 segment present in V-dependent channels [5]. Rather, these channels have evolved a high dependency on the binding to cyclic nucleotides to achieve the gate of the pore [6].

In the preceding paper [7], we evaluated structural aspects that allow us to better understand the role played by intrinsic flexibility of segment S3 in the basic aspect of the activity of ion channels: their ability of gating. We determined that segment S3 has two regions of important local flexibility: (1) a short sequence NxxD motif located at the N-half portion and (2) a short sequence at the beginning of the so-called *paddle* motif, around the half of S3, where the helix has a kink at Pro-322 (*Shaker* numbering), dividing it into two distinct helices (S3a and S3b). In *Shaker*-like channels, this *paddle* has high mobility during gating [8,9]. Notably, we found a good correlation between S3 flexibility indices and the sensitivity to temperature (measured as the Q**_10_** coefficient) so that if the channel exhibits high sensitivity to heat, local S3 helix become more rigid and *vice versa*. On the other hand, we also reported a good correlation between the degree of S3 flexibility and activation by mechanical stretching [7]. In the present study, we extend our analysis exploring the origins of the 6TM channels and we found that in one of the more ancient bacterial lineages (Aquificae) the only transport protein with significant similarity to members of the 6TM members of the VGIC superfamily corresponds to a putative potassium channel (kch) protein (ORF Aq1863) [10]. By sequence analysis, we found that this sequence has a residue identity of ~30% in comparison with CNG channels so, instead of belonging to the K_V_ family, kch protein rather could be considered as a member of the CNG-like channel family. In an effort to unveil the ancestral characteristics of one of the first 6TM channels, this finding prompted us to explore the contribution of the local flexibility present in the VSD and the PD separately, and compare them with the corresponding domains in different members of the CNG family, including several other prokaryotic ion channels, proton channels and voltage sensor-containing phosphatases (as models of the VSD). We also included several TolQ/PomA proteins, which we found exhibiting important sequence similarity with the typical V-sensor, as well as some 2TM channels, including members of the K**_ir_** family (as models of the PD). Our results highlight the role of protein flexibility in the evolution of CNG channels and suggest how the archetypal S4 segment in the VSD replaces Lys by Arg as the main charge carrier in voltage-dependent channels. In addition, we observe how the flexibility of the VSD decreases in modern lineages of such voltage-sensitive ion channels.

Therefore, in order to explore the ancestral nature of one of the first 6TM channels, we also generated a structural model of the kch protein, which we renamed here as the AqK channel, from the thermophilic bacteria *A. aeolicus* VF5, one of the earliest diverging prokaryotes known. Finally, considering this structural information as well as the flexibility analysis on different CNG channels, we decided to study the relationship between protein flexibility and ligand-dependency in this family of ligand-gated channels. To this aim, we compare the reported half-maximal effective concentration (EC**_50_**) for cAMP and cGMP as a function of the mean flexibility of the voltage-sensor domain in bacterial and eukaryotic CNG channels. Our results suggest that bacterial CNG channels are less flexible than their eukaryotic counterparts and almost fully dependent on cAMP while eukaryotic ones are mainly activated by cGMP and more flexible in comparison. Evidence also indicated that prokaryotic CNG channels are considerably voltage-dependent whereas eukaryotic channels have lost this attribute. Besides, analysis of sequence alignments from prokaryotic 6TM channels, voltage-sensing phosphatases, and proton channels, reveal significant similarity with TolQ and MotA/PomA orthologs from numerous Gram-negative bacterial genomes and Gram-positive archaeal hyperthermophilic methanogens such as *Methanopyrus*. These proteins are part of the Tol-Pal system, which plays an important role maintaining outer membrane integrity (TolQ) or as part of the ion motive force-dependent stator of the flagellar motor in Gram-negative bacteria (MotA/PomA). On the other hand, regarding the pore-domain, sequence comparison of bacterial inward rectifiers as well as prokaryotic 6TM potassium channels, including archaeal homologs, indicate that the increase in the flexibility profile in CNG channels is accompanied by a less conserved K**^+^**-selectivity signature sequence (TVGYGD). Overall, our analysis suggests that, in CNG channels, the flexibilities of the V-sensor and PD contribute to determining ligand affinity and ion permeation respectively. In addition, our results also indicate that, in the context of the origin and early evolution of life, first 6TM ion channels could have been highly flexible at the beginning and we speculate on the V-sensor evolution, which could have arisen from an ion potential-driven molecular motor. The role of the local flexibility in the VSD and the PD in gating is discussed.

## Material and methods

### Data assembly and local flexibility prediction

We assembled a dataset which includes protein sequences from different prokaryotes and selected eukaryotic organisms. The main criterion was to incorporate sequences from diverse phylogenetic groups, taking into account those cases where structural or electrophysiological data are available. With that initial dataset, we added other species from among early divergent microbes by BLAST-P. In total, 32 sequences were included in this study (4 from archaebacterial genomes, 19 from bacteria, and 9 from eukaryotes). The protein sequences from Archaea and their accession numbers are: KvAP from *Aeropyrum pernix* (Q9YDF8.1), TolQ from *Methanopyrus* sp. KOL6 (WP_088335072); MthK K**^+^**-channel from *Methanothermobacter thermautotrophicus* str. ΔH (AAB85995), and KcsA from *Methanothermobacter* sp. EMTCatA1 (BAZ98552). The sequences from bacteria include: AqK from *Aquifex aeolicus* VF5 (AAC07678); TolQ from *A. aeolicus* (NP_214364); TolQ from *Pedobacter* sp. Hv1 (WP_055133587); TolQ from *Parapedobacter indicus* (WP_090623292); TolQ *Olivibacter sitiensis* (WP_028296819); PomA from *Vibrio mimicus* (WP_000362402); LpcK from *Lyngbya* sp. PCC 8106 (WP_009782512); AmaK from *Arthrospira* sp. O9.13F (WP_111893854); TerK from *Trichodesmium erythraeum* (WP_011614153); MloK1 from *Mesorhizobium meliloti* (4CHV_A); LliK from *Leptospira licerasiae* (WP_008595756); SthK from *Spirochaeta thermophila* (WP_013313430); KcsA from *Streptomyces lividans* (P0A334); KirBac1 from *Burkholderia* sp. (WP_004534169); NaK from *Bacillus cereus* ATCC 14579 (NP_830482); MscL from *Escherichia coli* (STI81551); MscL from *Mycobacterium* sp. (WP_011742224), MscL from *Staphylococcus aureus* (WP_118848104), *a*nd MscL from *Methanosarcina acetivorans* (WP_011022264). The selected eukaryotes were: ciVSP from *Ciona intestinalis* (BAD98733); ciHv1 from *C. intestinalis* (NP_001071937); ehHv1 from *Emiliania huxleyi* CCMP1516 (005762299); kHv1 from *Karlodinium veneficum* (AEQ59286); mHv1 from *Mus musculus* (NP_083028); TAX-4 from *Caenorhabditis elegans* (CAB63418); *Shaker* K**_V_** channel from *Drosophila melanogaster* (CAA29917); CNGA1 from *Homo* (P29973), and CNGA2 from *M. musculus* (Q62398). Estimation of flexibility indices has been described in the companion study [7].

**AqK channel modeling and validation**

Protein modeling was performed with the complete (455 amino acids, including the putative cyclic nucleotide-binding domain, CNBD) and partial sequence (225 first residues, excluding the CNBD) of the potassium channel protein from *Aquifex aeolicus* VF5 (gi|2984143|gb|AAC07678.1|). The sequences were modeled using the Iterative Threading ASSEmbly Refinement (I-TASSER) server based on the *ab initio*/threading method [11]. Initial modeling was performed from all structure templates identified by LOMETS [12] from the PDB library. In both cases, the Ca**^2+^**-activated K**^+^** channel Slo1 structure (PDB ID: 5TJ6) was the template of the highest significance in the threading alignments (Z-score: 5.53 and 4.42; C-score: 1.48 and 0.36 for partial and complete sequences, respectively). From this target, I-TASSER generated a large ensemble of structural conformations and the top four three-dimensional models were examined. Among them, the best one, based in the highest confidence value, was selected to perform the subsequent tests(the confidence of each model was quantitatively measured by C-score and TM-score, respectively: 1.48/0.92 for the partial sequence and 0.36/0.76 for the complete sequence). Finally, the models were subjected to conjugate gradient energy minimizations using Amber 16 package with Amber ff99SB-ILDN protein force field. After generating the three-dimensional model, structure validation and stereochemical analysis were performed using different evaluations and validation tools: PROCHECK [13] and ERRAT online servers [14]. From these analyses, the stereochemical quality of both protein models shows those have high quality. The generated models were deposited in the Protein Model Database (PMDB) [15] with PMDB identifiers PM0081832 (partial sequence) and PM0081831 (complete sequence). 3D images were produced using the PyMOL Molecular Graphics System, v1.7.2.

### Online supplementary information

Supplemental material provides figures depicting flexibility profiles of V-sensors compared with diverse TolQ proteins and sequence similarity of the TolQ-like protein from *Aquifex aeolicus* VF5 (UniProt ID: O67795) as well as the flagellar motor protein PomA from *Vibrio mimicus* (UniProt ID: A0A1D8SCS3) with S2 to S4 segments in V-sensors. This information is available online.

## Results

### Evolution of the voltage sensor and the pore domain

Once the relationship among local S3 flexibility, voltage dependence, thermo- and mechanosensitivity was previously established [7], we decided to study the VSD and the PD modules separately. For this, we reasoned that, given that a large amount of phylogenetic studies have led to the hypothesis that 6TM potassium channels both in prokaryotic and eukaryotic cells derived from a simpler common precursor present in a primitive form of life, now extinct [16,17], a logical way to approach that precursor would be to study the potassium channels present in the oldest lineages of the Bacteria domain. *Aquifex* is a deep-branching hyperthermophilic chemoautotrophic bacterium restricted to hydrothermal vents and hot springs that make it an excellent biological model for studying the early evolution of life [18]. Consistent with the idea of potassium channels as the first members of the VGIC superfamily, we found two open reading frames related to potassium transport proteins in *A. aeolicus*. One of them, the putative protein at the locus Aq1863 has a significant sequence similarity to 6TM K_V_ and CNG channels [10] hereinafter called AqK. In Fig. Suppl. 1 the primary sequence (455 residues) of AqK was aligned with several prokaryotic members of the cyclic nucleotide-modulated ion channels previously characterized in bacteria [19–21]. AqK consists of six putative transmembrane segments, S1–S6, a canonical selectivity filter of K**^+^**-channels, TVGYGD, a potential voltage sensor at segments S1–S4 with five Arg residues in S4 and sequence similarity to the gating charge transfer center (CTC) previously described in segments S1–S3 of K**_V_** channels [22,23]. As expected, AqK also exhibits two important flexibility hotspots in its structure: (*i*) the NxxD and the AxxP *paddle*-like motif in S3 and (*ii*) the “gating hinge” in the middle of the S6 helix which is a highly conserved Gly residue, but, as in other cyclic nucleotide-modulated potassium channels, it lacks the swivel element (PVP motif) which has been implicated in regulating the gating machinery in K_V_ channels by allowing the lower half of the S6 to move [24].

**Figure 1.**
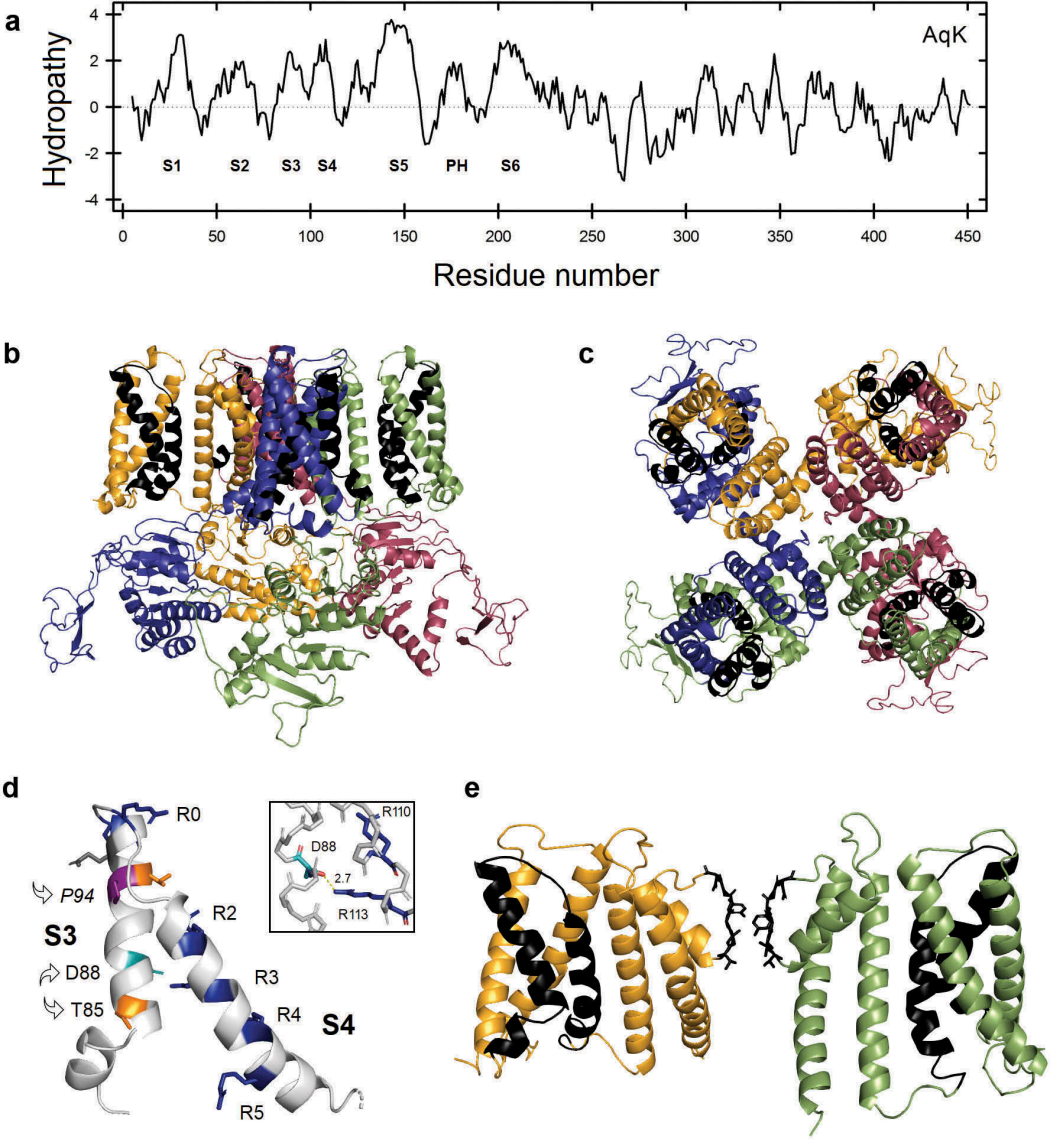
(a) Hydropathy plot of the AqK protein as calculated by the ProtScale program (web.expasy.org/protscale) according to the Kyte-Doolittle scale. (b – e) A three-dimensional model of the AqK channel. Protein structures are shown in α-carbon backbone cartoon representation, as viewed from the plane perpendicular to the membrane (b) or from the top of it (c). S3-S4 segments are represented highlighting important flexible side-chains in specific colors as in the companion paper [7]. Arrows show the NxxD and *paddle*-like motifs (d) or in black in two subunits for clarity, including the selectivity filter, also in black (e). Inset in panel d shows part of the gating charge transfer center highlighting Asp88, Arg110, and Arg113 (D88-R113 distance, 2.7Å).

As it has been reported for CNG and HCN channels from metazoan cells, the C-terminal end of AqK carries a cyclic nucleotide-binding domain (CNBD) connected by a C-linker. The full-length sequence of AqK was then compared with two previously characterized bacterial CNG channels, LliK, and SthK whose structures have been recently deposited in the Protein Data Bank (PDB codes: 5V4S and 6CJQ, respectively). LliK shares 33.3% sequence identity in 48 overlapping residues while SthK exhibits 25.2% identity in 115 residues, encompassing in both cases the pore domain and part of the VSD. In a first attempt to obtain a good model for this protein, we chose the structure of SthK as a phylogenetically proper template for the construction of a structural model for AqK. However, the Z-score for this preliminary model was rather poor (1.01 for the complete sequence and 2.02 for the partial one, encompassing only the transmembrane domain). In view of this, we evaluated several available structures and found that the Ca**^2+^**-activated K**^+^** channel, Slo1 (PDB ID: 5TJ6), was the best option to generate a good model for the AqK sequence (Z-score = 4.42 and 5.53 for the complete and partial sequences, respectively) (*see* Methods). Such as in the known structures of LliK and SthK, our AqK model reveals a non-domain swapped architecture in which each VSD associates with the PD of the same subunit as well as a kinked S3 segment, typical for this architecture, also present in TAX-4 (5H3O), HCN1 (5U6P), BK (5TJI), EAG1 (5K7L), and hERG (5VA2) channels. In each of these cases, this structural element is very close (about one helical turn) downstream of the NxxD motif (Figure 1).

**Figure 2.**
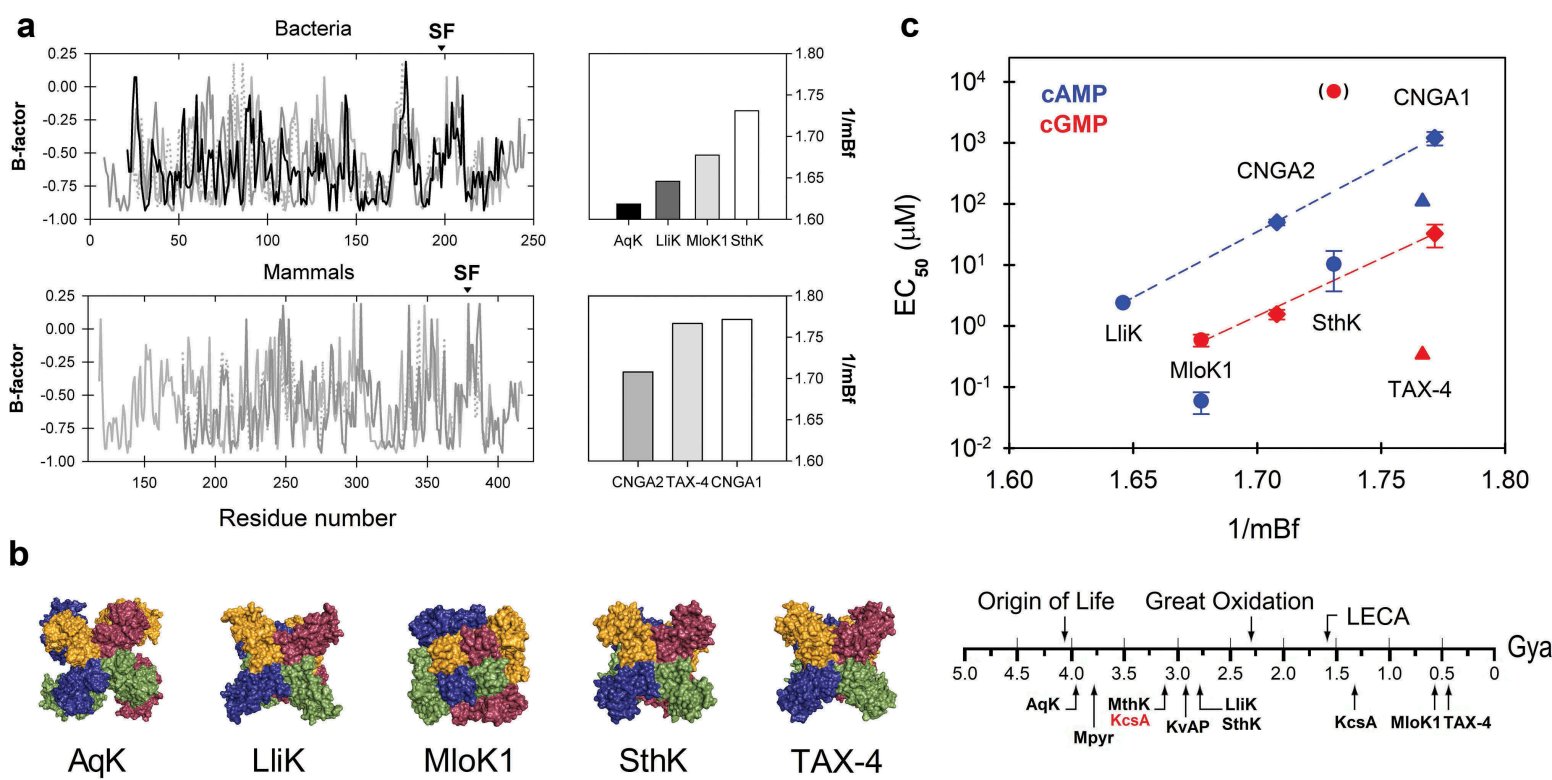
(a) S1-S6 flexibility profile in prokaryotic and eukaryotic CNG channels measured as the mean B-factor for the transmembrane core of the protein. SF, selectivity filter. (b) Top view structures for AqK, LliK, MloK1, SthK and TAX-4 proteins in a surface representation from the top with each subunit color-coded. (c) Ligand sensitivity measured as the half-maximal effective concentration (EC**_50_**) for cAMP (*blue*) or cGMP (*red*) as a function of the mean B-factor of the S1-S6 part from the respective protein (*up*) and time scale for ion channel evolution according to the first appearance of different forms of life where these proteins have been reported (*down*). The point in parentheses indicates that some authors report SthK activation by cGMP whereas others do not. The KcsA channel has been reported both in the archaeon *Methanothermobacter thermoautotrophicus* (3.2 Gya) as in *Streptomyces* sp. (1.3 Gya). Numbers are in billion years or giga-years ago (Gya). Mpyr: *Methanopyrus*; LECA: The last eukaryotic common ancestor.

Considering that AqK diverged very early in evolution [25], we set out to establish a relationship between the flexibility of these prokaryotic channels (AqK, LliK, MloK1, SthK) and their eukaryotic counterparts (TAX-4, CNGA1 and CNGA2) in terms of their dependence on cyclic nucleotides to be activated. To address this issue, we compared the reported half-maximal effective concentration (EC**_50_**) of cAMP and cGMP as a function of the mean flexibility of the 6TM segment for each ion channel. Figure 2 depicts the S1-S6 flexibility profiles for these proteins and indicates that the transmembrane core of AqK is, overall, comparatively more rigid than the one in LliK (from *Leptospira licerasiae*), MloK1 (*Mesorhizobium loti*) and SthK (*Spirochaeta thermophila*) which reinforces theory on protein rigidity as an adaptive strategy in thermophilic microorganisms [26,27]. On the other hand, eukaryotic CNG channels are very flexible proteins with important physiological roles. For example, TAX-4 is required for chemo- and thermo-sensation in *Caenorhabditis elegans* [28], whereas CNGA1 is mainly expressed in rod photoreceptors [29]. The transmembrane core of CNGA2 from olfactory neurons, although is more flexible in comparison to AqK, LliK, and MloK1, shows a profile slightly stiffer than SthK (Figure 2(a)). Then, we asked if at the structural level it could be recognizable some kind of pattern that would indicate the fact that the eukaryotic CNG channels tend to be in general more flexible than their prokaryotic counterparts. As Figure 2(b) indicates, we did not find any evident pattern revealing this trend, except for the fact that MloK1 exhibits a domain-swapped configuration, a wide outer pore [30], and the fact that in the only known structure for a eukaryotic CNG channel (TAX-4) the outer pore and the selectivity filter are even wider in comparison [5]. In any case, we detected an evolutionary trend in the response of these channels to their activators/modulators, *i.e*. the kind of cyclic nucleotide to which they respond. Interestingly, LliK, one of the more rigid CNG-like channels, has a high affinity for cAMP (EC**_50_** = 2.4 μM) but no dependency on cGMP has been reported [20]. On the other hand, SthK, which shows a flexibility profile comparable with the one present in their eukaryotic counterparts, is slightly less sensitive to cAMP (EC**_50_** = 10.4 ± 6.6 μM) but it shows a detectable sensitivity to cGMP [19,21]. On the contrary, eukaryotic CNG channels are activated mainly with cGMP and cAMP has less efficiency to do it [31,32] (Figure 2(c)). These results allow us to hypothesize that the high flexibility showed by eukaryotic CNG channels is linked to the preference for a specific ligand, *i.e*. cGMP. Thus, modern CNG channels in eukaryotes apparently increased in flexibility whereas replaced their dependence on cAMP by cGMP, besides they lose both their voltage dependence and their K**^+^**-selectivity, becoming essentially nonselective [6]. Future experiments, including ancestral protein reconstruction, molecular dynamics simulations, and molecular modeling, could help to test this possibility.

The rigid nature of the transmembrane core of AqK in relation to other prokaryotic and eukaryotic CNG channels, as well as the apparent trend to lose voltage sensitivity whereas they increase their intrinsic flexibility – acquiring a full dependence on cyclic nucleotides – led us then to ask the following question: Was the original voltage sensor also intrinsically rigid for the vast superfamily of VGICs prior to the appearance of AqK? The theoretical framework regarding VGICs evolution propose an ancestral fusion event between a 4TM voltage-sensor module and a 2TM pore module [3]. This has been proposed due to both kind of proteins also exist as independent functional units: the voltage-gated proton channels and the voltage-sensing phosphatases on one hand [33,34] and, on the other hand, the bacterial channels such as KcsA from *Streptomyces lividans* and the inwardly rectifying K**^+^**-channels [35]. With this in mind, in order to examine and try to better understand the relationship between the intrinsic flexibility and the “voltage-to-ligand” transition, a comparative analysis of the V-sensor module was performed. For this analysis, protein sequences of several VSDs from basal organisms were contrasted with sequences from modern lineages.

Thus, we decided to include in the analysis several Hv1 and VSPs homologs present in lower organisms since they could be the closer representatives of the ancestral VSD [36,37]. In addition, by searching Hv1 orthologs in the *A. aeolicus* genome, we found a small membrane protein (204 residues) corresponding to MotA/TolQ, which has been characterized as part of the stator complex in flagellar motors of several bacteria [38]. TolQ and their homologs (MotA, ExbB) are thought to be a “proton pore” involved in converting the proton-motive force (p.m.f.) into flagellar rotation [39]. The MotA/TolQ/ExbB family groups integral membrane proteins with scarce sequence identity but important structural and functional similarity that presumably form proton channels in hetero-complexes. These proteins use the p.m.f. either to generate rotational motion in the flagellum (MotA) or to stabilize the outer membrane integrity (TolQ) as well as to transport large molecules across it (ExbB) [40]. Likewise, these proteins also share sequence similarity with PomA, which are also part of the flagellar motor complex. These membrane proteins, instead of forming proton channels, form sodium channels, converting Na**^+^** fluxes to the rotation of the flagellum and are controlled by osmolality and pH [41,42].

**Figure 3.**
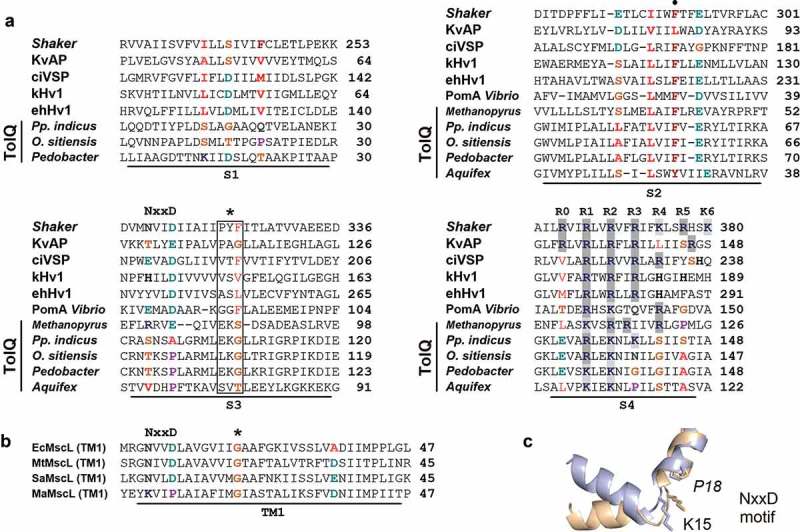
The similarity exhibited by flagellar motor complex proteins and V-sensors. (a) Sequence alignment of gating pore residues in several VSDs, TolQ, and PomA. (b) Alignment of TM1 segment in selected MscL proteins. In both cases, sequences were aligned using Clustal-W. The asterisk over the sequence alignment depicts the location of Gly-26 which is an important residue involved in gating, apparently conserved in the VSD as a point of high local flexibility. The black circle indicates the highly conserved aromatic residue in S2 which is crucial for the function of the V-sensor (c) Close-up view and NxxD-like side chain superposition of MaMscL wild-type in closed (silver), and expanded state (gold) showing the pivot point around which TM1 tilts under membrane tension. As proline is an “α-helix breaker”, it is depicted in italics.

Therefore, protein sequences from the VSD present in KvAP (*Aeropyrum pernix*, Crenarchaeota), ciVSP (*C. intestinalis*, Ascidiacea), kHv1 (*Karlodinium veneficum*, Dinoflagellata), ehHv1 (*Emiliana huxleyi*, Haptophyta) and the *Shaker* K**^+^**-channel (*Drosophila*) were compared with flagellar motor proteins TolQ and PomA from *A. aeolicus*, several members of the Phylum Bacteroidetes, Proteobacteria and *Methanopyrus* (Euryarchaeota). Notably, this comparison indicates that there is a number of regions that shows common elements of the archetypical VSD: (1) the specific counter charges described in the gating pore [23], (2) a flexible NxxD-like motif described in the S3 segment, (3) a flexible *paddle*-like motif [8], and (4) the presence – in some cases – of several positive charges described in the S4 segment of VGICs with some degree of conservation, *i.e*. the typical alternating motif of a positively charged residue followed by two hydrophobic residues [4,43]. Some of these specific positions, depicted in Figure 3, are frequently occupied by small or very flexible residues in organisms that diverged early in the evolution. One clear example is the *paddle* motif (PYF in K_V_ channels) whose counterpart in TolQ-like orthologs (EKG motif) is intrinsically even more flexible. Similarities were also evident between the first TM helix of MscL proteins and segment S3 from *Shaker*, KvAP, and the TolQ-like sequence. Most importantly, there is a well-conserved Gly residue in MscL (G22 in *Mycobacterium tuberculosis*; G26 in *E. coli*) and an equivalent residue in TolQ-like proteins, which is at a similar distance from the putative NxxD motif in those proteins (Figure 3(b)). In MscL, G26 constitutes the constriction point at the lumen of the closed channel and is thought to contribute stabilizing the open conformation [44]. Our results indicate that this region is particularly well conserved between distant proteins, suggesting for an important functional and/or structural role for this sequence motif and also establishing a link between mechano- and voltage-gating. This reinforces a previous hypothesis that suggest those processes could share a common evolutionary origin [45].

**Figure 4.**
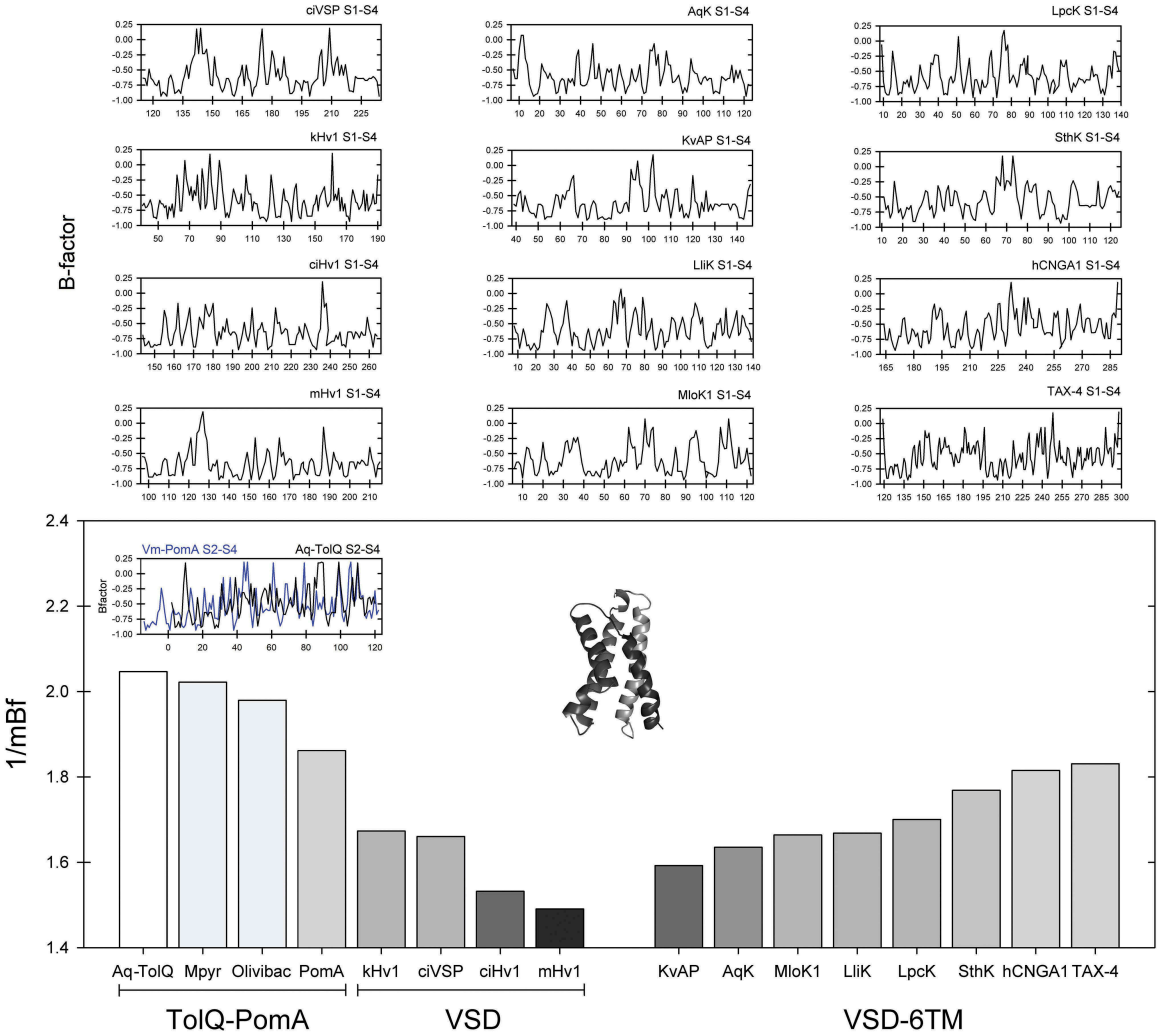
Comparisons of the mean B-factor profiles from stator proteins TolQ, PomA, and the voltage sensors from Hv1, ci-VSP, KvAP, and diverse CNG ion channels. The profiles for flagellum stator proteins (TolQ/PomA) are highly flexible, which is consistent with their topology. Nearly all positively charged amino acid residues of TolQ/PomA proteins, including those with analogous to the corresponding residues in S4 from true V-sensors, are located in the cytoplasm. This agrees with the so-called positive inside rule, stating that cytoplasmic membrane proteins contain a surplus of positive charges at the inside of this membrane [73]. Inset represents the VSD from AqK.

Once sequence similarity between these flagellar motor proteins and the VSDs was established, we also quantified the intrinsic flexibility of TolQ-like proteins and contrast it with the one present in the VSD. Selected members of the VGIC superfamily, present in basal and modern descendants, were then compared. Our results clearly indicate that TolQ-like proteins are comparatively much more flexible than any of the proton channels, VSP or the V-sensor from K**_V_** and CNG channels (Figure 4). This is consistent with the topology of TolQ in *E. coli*, which consists of three membrane-spanning regions with a large cytoplasmic loop (98 residues) after the first TM segment and a long cytoplasmic C-terminus. Notably, insertion of the hairpin loop formed by the second and third membrane-spanning regions of TolQ is dependent on the membrane potential [46]. These results also suggest that first proton channels present in lower eukaryotes (Protista) were comparatively more flexible than those present in basal metazoans (ascidians) and that the evolutionary trend, at least in the Hv1 family, apparently was to increase the rigidity of these proteins, judging by the profile found in mammalian Hv1 channels (Figure 4). On the other hand, comparison between voltage sensors in 6TM channels, including those present in one of the first voltage-dependent K**_V_** channels such as KvAP (from the archaeon *Aeropyrum pernix*) as well as in prokaryotic and eukaryotic CNG channels, indicates that in modern lineages the trend in this family was to increase the flexibility of the V-sensor, while their dependence on cyclic nucleotides also increased. Concomitantly, their voltage dependence disappears. Although this scenario is consistent with various experimental data reported elsewhere [19,20,29,31,47], more research is needed to confirm this hypothesis.

**Figure 5.**
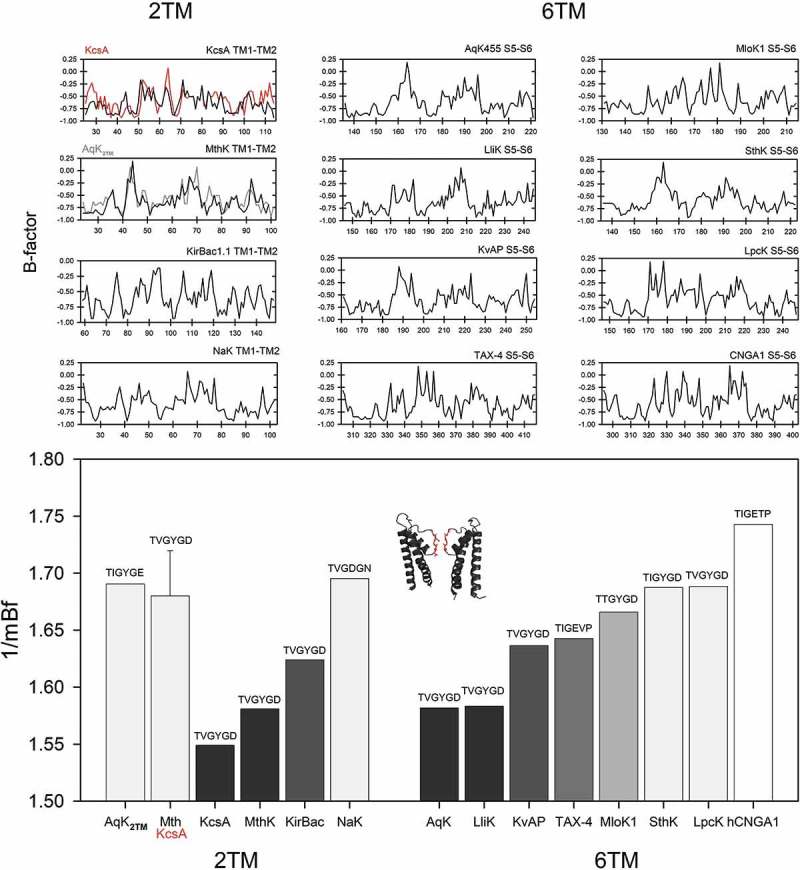
Comparisons of the mean B-factor profiles from the pore domain in 2TM and 6TM channels including voltage-dependent and CNG proteins. The sequence for the selectivity filter is shown at the top of the corresponding bar. Results show that while the ancient 2TM KcsA channel from *Methanothermobacter* (red) had a quite flexible pore, its modern counterpart (*Streptomyces*) is significantly more rigid. This character was lost once channels become nonselective (NaK channel from *Bacillus cereus*) and become more and more dependent on ligand (as in CNG channels). Inset represents the PD from AqK.

Finally, our last aim was to quantify and compare the flexibility index of the pore domains in 2TM and 6TM channels, including the archetypical KcsA from *Streptomyces lividans* and the one present in *Methanothermobacter thermautotrophicus*, which has diverged much earlier in evolution [25]. In this analysis, we also included the Ca**^2+^**-dependent MthK channel from the same species and the one we found in the *Aquifex* genome, which we named as AqK**_2TM_**; the inward rectifier K**^+^**-channel from *Burkholderia* (KirBac); and the nonselective cation channel NaK from *Bacillus cereus*. Interestingly, AqK**_2TM_** shows sequence similarity with MthK: 24.6% identity in 329 residues overlaps but also a very high flexibility profile, comparable to the one exhibit by the KcsA ortholog present in *M. thermautotrophicus*. On the contrary, the flexibility profile in the PD of MthK is similar to the one from AqK or LliK, which are considerably more rigid. It is also notorious that the two compared KcsA channels show very different profiles, being that belonging to the oldest lineage (*M. thermautotrophicus*), significantly more flexible than the modern one (*S. lividans*). The flexibility profile associated with a modern nonselective channel (NaK), present in *B. cereus*, which is estimated to have diverged very recently in evolutionary terms (~1) Gya according to [25], is also very high compared to the rest of the modern 2TM channels (Figure 5).

**Figure 6.**
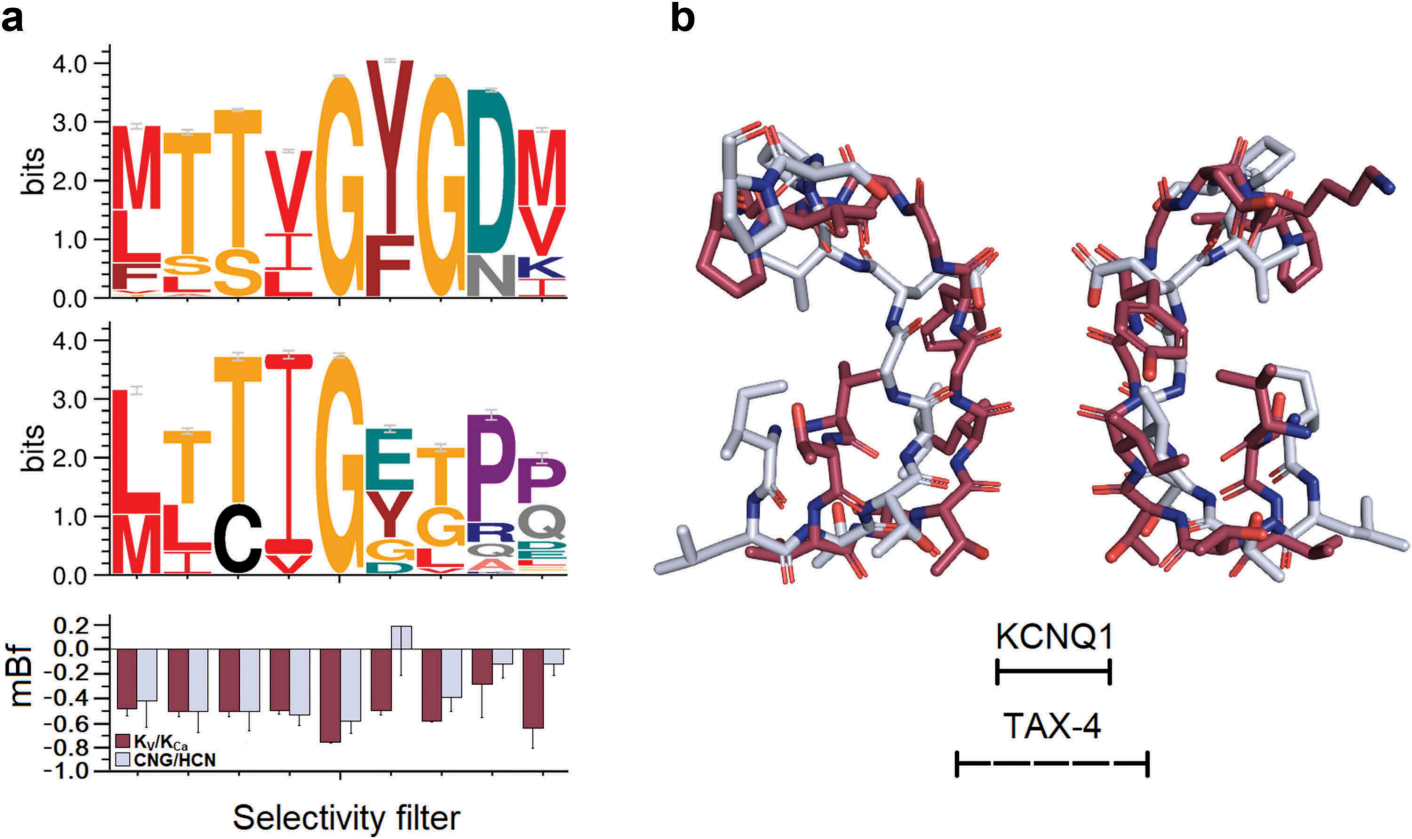
The flexibility of the selectivity filter in voltage-gated and ligand-dependent ion channels. (a) Sequence logos of the K**^+^**-channel signature sequences for K_V_1–4, K_V_7, K_V_10–12 and K**_Ca_**1–5 channel proteins (*up*) and the selectivity filter present in the CNG-AB/HCN1–4 clades (*middle*) from metazoan genomes. The comparison of the predicted mean B-factor for these sequence motifs are shown (*bottom* plot) in *marron* (G-Y-G-D motif) or *gray* (G-E/Y-T/G motif). (b) Overlay of the Kv7 (KCNQ1, *marron* backbone) and the TAX-4 selectivity filters (*gray* backbone) pore domain structures. The cartoon depicts two diagonally oriented subunits, with front and back subunits omitted for clarity. The side chains correspond to equivalent positions as in the sequence logos. K_V_7.1: PDB ID 5VMS; TAX-4: PDB ID 5H3O.

Regarding the PD present in 6TM channels, we analyzed the following sequences: AqK, LliK, KvAP, MloK1, SthK, LpcK (*Lyngbya* sp. PCC 8106), TAX-4 and human CNGA1. Overall, flexibility in the VSD in these proteins is higher than the one corresponding to their corresponding PD, which is consistent with the conformational changes associated with voltage gating performed by that domain. As in the case of the VSD, it should be noted that PD flexibility increases as the channel depends more and more on its activation with cyclic nucleotides and that the K**^+^**-selectivity is directly associated with the presence of the highly conserved rigid motif, TVGYGD signature sequence present at the selectivity filter (Figure 6). The relevance of these observations is yet to be resolved but, taken together, our analysis suggests the possibility that the VSD, represented by TolQ-like proteins, was very flexible at the origin. On the other hand, the primordial PD, whose modern representatives are the KcsA and MthK orthologs from *M. thermautotrophicus* and *A. aeolicus* respectively, was also very flexible in nature.

## Discussion

According to Yu and Catterall [1], the similar functional elements of the VGIC superfamily can be divided into three complementary aspects: (1) ion conductance (permeability/selectivity), (2) pore gating, and (3) regulation (modes of activation/inactivation and deactivation) in response to changes in membrane voltage, signaling molecules (ligands), or both. The modular nature of the voltage-dependent ion channels, its presence in primitive microorganisms such as *Aquifex* and *Methanothermobacter*, and the vast diversity of members of the VGIC superfamily indicates an early origin for this kind of proteins and the extensive exploration of a basic architecture. How did the structural domains (VSD and the PD) of this large superfamily arise during life evolution? Some insight can be deduced by comparing ion channels in deep-branching prokaryotic species. Many bacteria and some virus have 2TM K**^+^**-channels resembling inward rectifiers, and some early bacteria have 6TM voltage-gated K**^+^**-channels. On the other hand, both Hv1 proton channels as the voltage-sensing phosphatase are considered proteins with different physiological roles derived from a common functional unit [34,36,37]. In this scenario, it is conceivable that first 6TM members of this superfamily were 2TM K**^+^**-channels fused to an early S1-S4 voltage sensor as both modules are independent functional units [1,3]. This hypothesis has been experimentally tested by the fusion of the VSD of *C. intestinalis* phosphatase to the 2TM viral channel K**_cv_** creating a functional voltage-gated, outwardly rectifying K**^+^**-channel [2] or by conferring voltage dependence to V-insensitive channels [48].

Considering that *A. aeolicus*, a thermophilic bacteria, is one of the earliest diverging prokaryotes known [25] and since its complete genome has been annotated [10], we reasoned that in order to gain insights about the nature of the first 6TM K**^+^**-channel, the study of the AqK protein would contribute to establishing a framework to evaluate this theoretical fusion event and how this superfamily has evolved. We found two sequences in the genome with important similarity to K**^+^**-channels: one of them (AqK, 455 residues) strongly remembering a CNG channel (GenBank: AAC07678.1) and the other one (AqK**_2TM_**, 368 residues), is a putative MthK ortholog. Both the primary sequence of AqK, as well as its intrinsic amino acid hydropathy, predicts helix insertion into the membrane. With this in mind, we generated a homology model in order to structurally study important aspects of its assumed biological function (Figure 1). In a future investigation, we will follow the same approach to characterize AqK**_2TM_** and perform deeper evolutionary studies.

AqK shows 25.2% identity in 115 residues overlapping segments S4 to S6 when it is compared with SthK, although the degree of similarity exhibited by these proteins is not shared at the level of their intrinsic flexibilities. Compared to SthK, the S1 to S6 profile of AqK is considerably more rigid, which make sense if the natural habitat of *A. aeolicus* is considered; *i.e*. near underwater volcanoes or hot springs at temperatures between 85–95°C [10,18]. In comparison, SthK was isolated from *S. thermophila* whose optimum growth temperature range has been described between 66–68°C [49]. LliK, another prokaryotic cyclic nucleotide-gated ion channel from *L. licerasiae*, is also more rigid than SthK but slightly less than AqK so it requires physiological temperatures for an optimal performance. If we consider these data in temporary perspective, it is evident that *Aquifex* diverged from the last universal cell ancestor (LUCA) little less than 4 billion years ago and the Spirochaetales did it ~1.1 million years later [25]. Our analysis indicates that the VSD in eukaryotic CNG channels is very flexible but their more ancient prokaryotic counterparts are comparatively more rigid, sharing a common non-domain swapped architecture. Biophysical studies of LliK, SthK and MloK1 have also shed some light on the cyclic nucleotide dependence for gating in these proteins, indicating that cAMP is clearly preferred over cGMP [19,20,47,50]. Instead of, in the case of eukaryotic channels (TAX-4, CNGA1/A2), activation is carried out preferentially in the presence of cGMP [31,51].

The interesting pattern depicted in Figure 2 could suggest that in the origin, first CNG channels had adenine nucleotides more available for their activation instead of guanine ones, which is consistent with the ease with which adenine is synthesized in prebiotic conditions [52]. Although we did not perform an analysis of the CNBD, data shown in Figure 2 indicate that CNG channels with flexible transmembrane domains clearly prefer guanine cyclic nucleotides. If the flexibility of this domain is comparable to the one in the CNBD, this could indicate that cGMP can be better accommodated into a flexible environment than into a more rigid one. This is consistent with the fact that cGMP has a weak but detectable agonist effect on the activation of the flexible channel SthK [47], but in the rigid one, *i.e*. LliK, this has not been achieved [20]. MloK1, however, is strongly activated by cGMP but even in this case, it prefers cAMP [50]. This apparent inconsistency could be explained if one considers that only in this channel, a domain-swapped arrangement has been reported [30] and this organization implies a long S4-S5 linker (15 residues in MloK1). As the S4-S5L has high internal flexibility playing a clue role coupling the V-sensor to the channel’s gate in K**_V_** channels [53], it has been associated with a high degree of mobility [9,54]. Therefore, we argue that, albeit MloK1 is intrinsically rigid in composition, its domain-swapped architecture facilitates important mobility during gating, in addition to certain specific interactions which could be favored by this arrangement.

The trend to increase the intrinsic flexibility into the CNG family prompted us to test if the rigid profile of the VSD from AqK can represent the original nature of the first V-sensor into the whole VGIC superfamily. As mentioned earlier, both Hv1 proton channels and V-sensing phosphatases are evidence that the V-sensor has evolved as a functional unit. However, prokaryotic counterparts for these proteins have not been reported [34,37]. On the other hand, there is evidence which suggests that the V-sensor from Ci-VSP (Ci-VSD) might represent an evolutionary intermediate between the V-sensor in voltage-gated ion channels and proton channels [36].

In search of the primeval VSD, we found intriguing sequence similarity between the canonical VSD in VGICs and prokaryotic TolQ proteins. TolQ from *E. coli* is a 230-residue integral cytoplasmic protein which span three times the membrane and whose role is to import group A colicins during phage infection, cell division as well as the maintaining of the integrity of the outer membrane. Which is interesting in the context of the V-sensor evolution is the fact that membrane insertion of TolQ is not dependent on the Sec system, but it requires the membrane potential [46]. Previous sequence comparisons indicate that the TolQ-TolR complex share important similarity with the MotA-MotB complex which uses the p.m.f. to propel the flagellum [40]. TolQ from *A. aeolicus*, which diverged very early in evolution, *i.e*. ~3.9 Gya, has a sequence similarity of 22.3% and an amino acid identity of 11% with the proton-gated Hv1 channel from *Karlodinium veneficum* (Dinoflagellata). This channel is perfectly selective to protons and recent findings suggest that Hv1 channels in these basal organisms have functions separate from only its role in bioluminescence and thus it has been suggested that Hv1 has an ancestral function in dinoflagellate biology [55]. Considering this similarity between two distant proteins but sharing a common function, which is to allow proton fluxes, we asked if this sequence similarity is conserved in the early-branching archaeon *Methanopyrus*. Our findings indeed demonstrate that TolQ from *Methanopyrus* is slightly more similar to kHv1 than the *A. aeolicus* ortholog (25.8% similarity). In order to obtain more insights on this point, we also tested modern descendants of TolQ in other bacteria and then try to extract a relevant conclusion. Hence, the sequence comparison between several TolQ homologs from *Pedobacter, Parapedobacter* and *Olivibacter* (~2.0 Gya) and the flagellar motor protein PomA from *Vibrio mimicus* (~0.8 Gya) reveals an interesting pattern where the number of positively charged residues (usually Lys) at the S4-like segment is gradually replaced by Arg. Notably, in true V-sensors, for instance, in kHv1, Ci-VSP or the *Shaker* K**_V_** channels this last amino acid is preferred, probably because their unique hydration properties which provide better efficiency to facilitate the V-sensor motion [56]. Moreover, the guanidinium group of Arg allows interactions in at least three possible directions, which enables its side-chain to form a larger number of electrostatic interactions in comparison to Lys, and favors more rotameric conformations due to its size, side-chain flexibility and higher capability of carrying charges [57,58]. This is also consistent with the study of Berezovsky et al. [59] which clearly demonstrate that in hyperthermophilic genomes a greater proportion of positively charged residues is almost entirely due to Lys but not Arg since MD simulations show that Lys has a much greater number of accessible rotamers than Arg and, in consequence, those proteins are overall more flexible.

The possibility that TolQ represents an ancestral form of the V-sensor should be taken, however, with caution. The sequence similarity we found is not too high, even though certain key residues, critical for the function of the V-sensor, seem to be well preserved. Such is the case of the Tyr27 which in the rest of TolQ orthologs we analyzed is a Phe (Figure 3), *i.e*. a large and rigid side chain. In *Shaker* K^+^-channels that position is critical. The highly-conserved Phe290 at S2 has been described as a molecular clamp helping the movement of S4 at the gating pore or the so-called charge transfer center, CTC [22,23]. Other important residues in this CTC are for example Glu293 and Asp316 (*Shaker* numbering) which, according to Papazian et al. (1995), interact with Lys374 and Arg377 in S4 during gating [60]. In addition, Glu293 (*Shaker*) is also well-conserved in TolQ proteins, around the same distance; Asp316 (at the NxxD motif) is less-conserved but also present in some PomA proteins. In the TolQ protein from *Aquifex* there is a Pro residue at the equivalent position; this residue has also been described in very early mechanosensitive MscL orthologs, particularly in the archaeon *M. acetivorans* (~3.1 Gya) where it plays an important role favoring channel expansion under high lateral tension [61] (Figure 3(C)). The last interesting similarity is the *paddle* motif, which is not conserved at the primary sequence level in V-sensors and TolQ proteins – indeed it is almost exclusive of *Shaker*-like channels – (Figure 3(a), *box*) but whose high flexibility profile is quite similar in those proteins (Fig. Suppl. 2).

Also interesting is the fact that in TolQ as well as in their homologs (MotA and PomA for example) there have been described several mutants where swarming motility is impaired, or the cell envelope integrity has been altered [62-65]. Notably, some of these mutations are situated in segments showing similarity with S2/S4 and correspond to Phe290, Glu293, Lys374 and Arg377 in the *Shaker* K**_V_** channel (Fig. Suppl. 3). In *Shaker*, the closed conformation is stabilized by salt bridges formed between Lys374 in S4 and Glu293 as well as with Asp316 in S2 and S3 segments, respectively [60,66,67]. In Hv1 proton channels, this position (K374 or ‘R4’) is occupied by a neutral Asn (N210 in mouse Hv1) or a basic residue (H183) in kHv1, and also in this case, evidence indicates that region around this position, plays a key role in the function of such V-sensor, by forming the proton conduction wire during permeation [65]. On the other hand, the conserved F290 (*Shaker*) located at S2 interacts with the gating charges (R1 to R4) in S4 [22]. Similarly, in Hv1 proton channels, the equivalent position (F150 in human Hv1; F119 in kHv1) demarcates inner and outer aqueous vestibules during gating [33]. Besides, many different mutations in K**_V_** channels have been identified resulting in the so-called “omega current” [66]. In *Shaker*, S4 mutants (R1H) support proton conduction at negative voltages (*i.e*. at resting). This current can be carried also by a variety of other ions including K^+^, Cs^+^ and even large organic ones [66,67]. On the contrary, in TolQ and MotA proteins, the proposed aqueous proton channel forms in association with TolR at the Q2/Q3/R or MotB at the A3/A4/B TM interfaces throughout intra- and intermolecular interactions [68–70]. Again, although these two situations are different, they show something in common, *i.e*. the fact that ion flux is favored through helical interfaces where specific residues interact and, importantly, size matters. Both in the VSD as in the TolQR and MotAB heterodimers, small side chains in specific positions are preferred to open the ion flux pathway. There are clear parallels in certain key residues and sequence motifs in V-sensors and TolQ-like proteins, so it is tempting to speculate about some kind of evolutionary link between these apparently distant proteins, and then more research becomes necessary to test this hypothesis.

Regardless of the weak similarity we found between TolQ/PomA proteins and V-sensors, membrane topology is completely different: (1) unlike what is observed in V-sensors, TolQ has the N-terminus located in the periplasm, only in BK channels a similar arrangement has been reported by the additional S0 TM segment so that its N-end faces the extracellular side [71]; (2) TolQ contains only three (Q1-Q3) TM segments, instead of four (S1-S4) in V-sensors [40,72]; (3) in the *Aquifex* ortholog (AqTolQ) only segment Q1 correspond to S2 in the canonical V-sensor whereas segments showing similarity with S3 and S4 are located in the long cytoplasmic loop between segment Q1 and Q2 (Fig. Suppl. 3a); (4) in PomA, a TolQ homolog from *Vibrio* has, however, four (A1-A4) TM segments and again in this case, sequence similarity to segments S2 to S4 are located between the first two TM segments and mainly in the long cytoplasmic loop between A2 and A3 segments (Fig. Suppl. 3b). All these differences are however very interesting from an evolutionary perspective. If there is a link between proteins of the flagellar motor from certain thermophilic microorganism and the appearance of the V-sensor, several questions emerge: (1) Were the positively charged residues in V-sensors, originally regions of high local flexibility involved in torque generation in a primordial stator protein?; (2) As topology in membrane proteins is controlled primarily by hydrophobicity, length of TM helices, distribution of positively charged residues in the loops, as well as the electrochemical potential [73–75], was the appearance of the canonical V-sensor the result of a topological reconfiguration of a primordial stator protein from the bacterial flagellum? Why did this happen? Many biological processes are coupled to ion gradients throughout lipid membranes, so ion fluxes at the interface of TM helices generate mechanical energy to drive diverse processes. Is not unreasonable suspect that, in order to take advantage of this energetic potential, the primordial V-sensor acquired ion selectivity by the association with a 2TM K**^+^**-selective pore and finally developed gating properties to accomplish specific physiological roles; (3) Why the CNG-like channels are so ancient and, apparently, the first in appear besides Ca**^2+^**-modulated 2TM K**^+^**-channels?; (4) Assuming a fusion event between a 4TM VSD and a 2TM PD, why first 6TM CNG-like channels, in addition to responding to voltage, are highly sensitive to the activation by cyclic nucleotides, in particular cAMP?; and finally (5) When did the 6TM channels first appear? Our results suggest that the V-sensor and the pore domain were conceivably very flexible at the origin of life, which is consistent with recent findings on protein evolution [76,77]. Much of this flexibility was conserved in the family of CNG channels and diverged in very different ways within the VGIC superfamily, sharing a similar molecular architecture and developing diverse physiological properties.

## Conclusions

In 6TM channels, modularity has been exploited several times in evolution. The sequence-to-flexibility analysis we performed in this study indicates that both the V-sensor and the pore domain modules were probably highly flexible at the beginning of life. However, in earlier prokaryotic CNG channels this flexibility was apparently reduced, probably as an adaptation to high-temperature habitats. This rigid character seems to be associated with high selectivity to K**^+^** ions and to strongly preferring cAMP instead cGMP to activate the protein, as in the case of LliK, MloK1, and probably also in AqK channels. In modern CNG channels, however, flexibility significantly increases, linked to the fact that they lose ion selectivity and favoring activation by cGMP instead cAMP. This is the case of TAX-4 and CNGA1/2 channels. Our analyses also indicate that the VSD exhibits sequence similarity to bacterial H^+^- and Na^+^- driven flagellar motor proteins as well as the TM1 segment in ancient MscL orthologs. These findings reinforce a previous proposal suggesting a common origin for mechano- and voltage-sensing and open interesting questions regarding to the V-sensor evolution. Finally, our AqK model from the hyperthermophile *A. aeolicus* provides important structural information to evaluate and contrast a very ancient 6TM channel with modern counterparts of the VGIC superfamily and to understand the evolution of CNG channels.

## Dedication

This work is dedicated to Chem. Guillermo Krötszch (1952–2019), an amazing philosopher, scientist, and friend. 500 years after Leonardo …Our partner in time.
