# Supplementary figures and images for: Voltage vs. Ligand II: Structural insights of the intrinsic flexibility in cyclic nucleotide-gated channels
Source: Channels (Austin). 2019 Sep 25;13(1):382–99. doi: 10.1080/19336950.2019.1666456 (PMC6768053; doi:10.1080/19336950.2019.1666456)

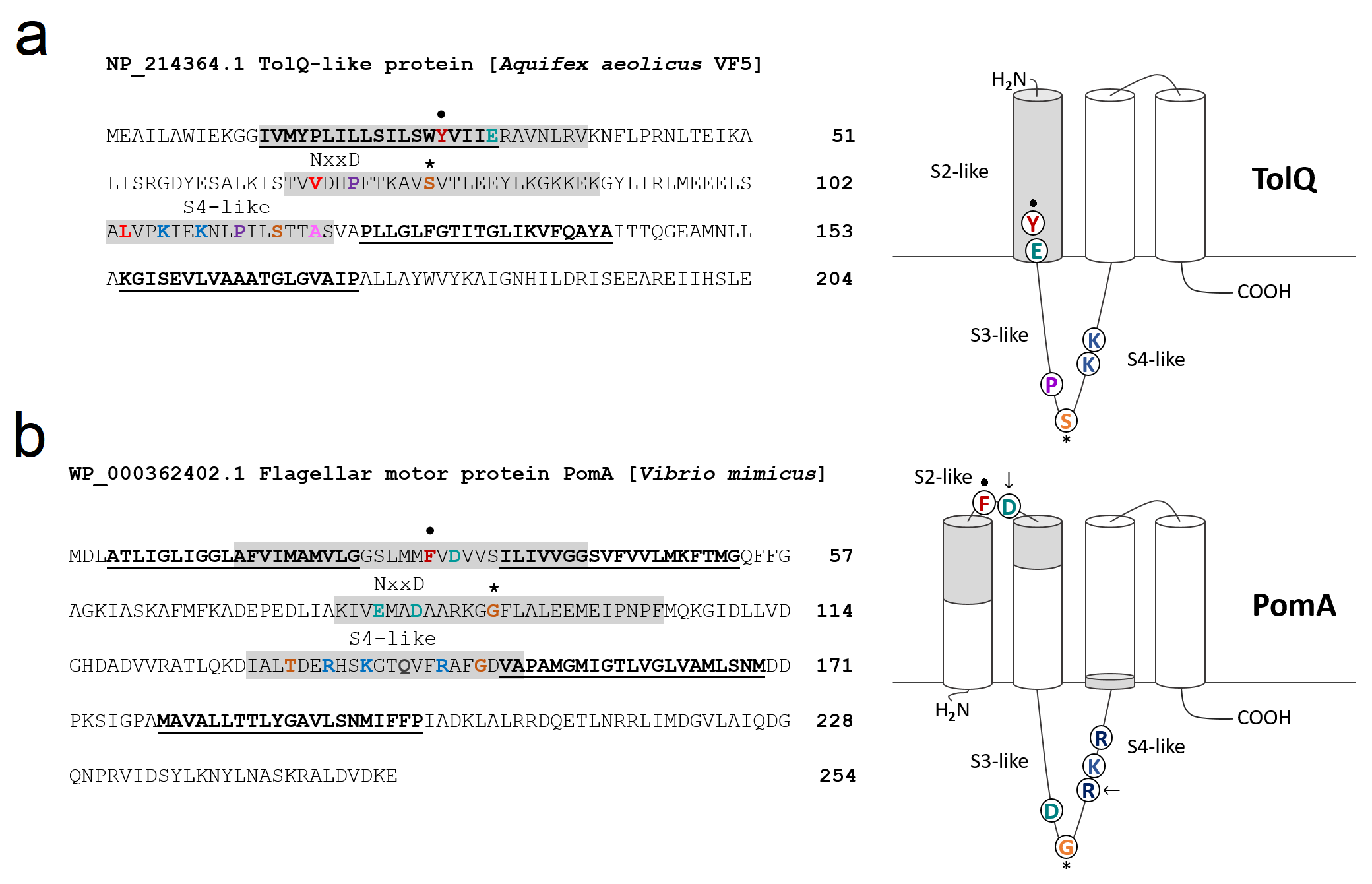

Supplement: Supplemental Material [file kchl-13-01-1666456-s001.png]

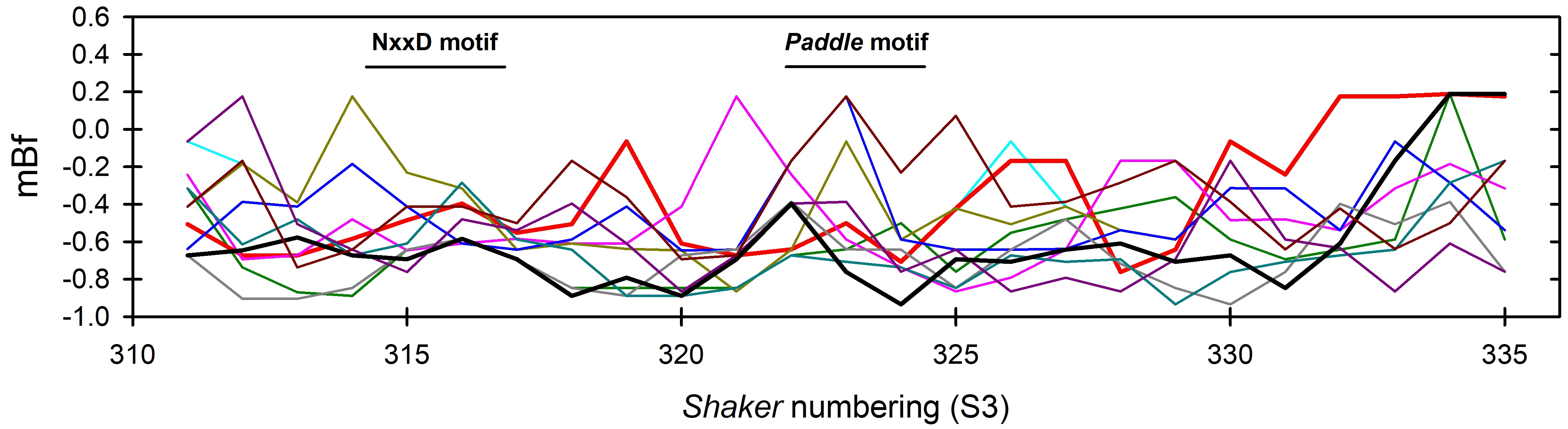

Supplement: Supplemental Material [file kchl-13-01-1666456-s002.jpg]

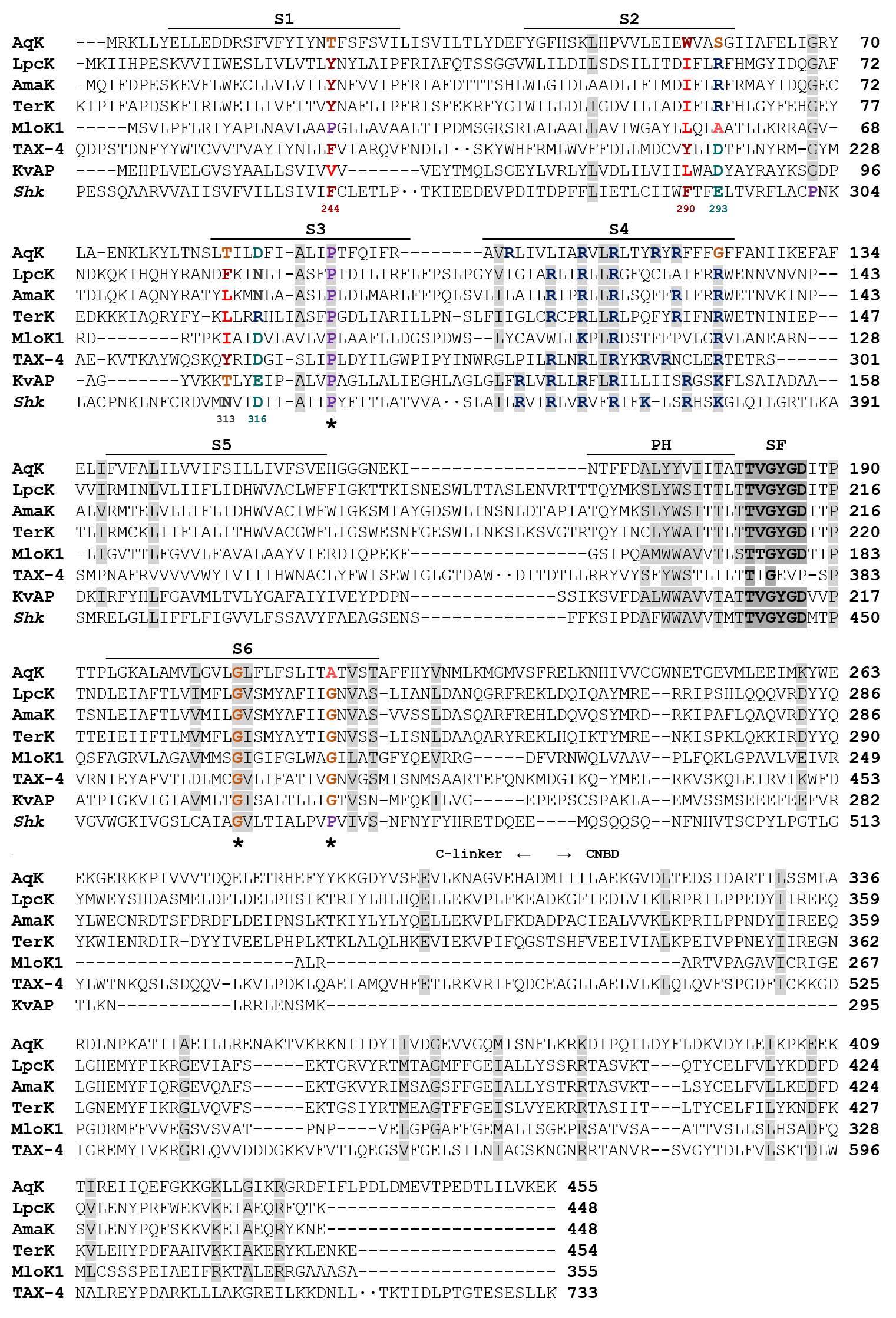

Supplement: Supplemental Material [file kchl-13-01-1666456-s003.png]
