# Supplementary material for: Voltage vs. Ligand II: Structural insights of the intrinsic flexibility in cyclic nucleotide-gated channels
Source: Channels (Austin). 2019 Sep 25;13(1):382–99. doi: 10.1080/19336950.2019.1666456 (PMC6768053; doi:10.1080/19336950.2019.1666456)
Supplement: Supplemental Material [file kchl-13-01-1666456-s004.docx]

***Supporting Material***

**Fig. S1.** Sequence analysis of the prokaryote cyclic nucleotide-modulated channels AqK channel from *Aquifex aeolicus*, LpcK (*Lyngbya* sp.), AmaK (*Arthrospira* *maxima*), and TerK (*Trichodesmium erythraeum*; the eukaryotic cyclic nucleotide-gated channels MloK1 (Mesorhizobium loti) and TAX-4 (*Caenorhabditis elegans*); and the voltage-dependent potassium channels KvAP from the hyperthermophilic archaeon Aeropyrum pernix, and *Shaker* (*Shk*) K**^+^**-channel from *Drosophila*. Asterisks indicate points of important local flexibility.

**Fig. S2.** Flexibility profile in segment S3 and TolQ proteins. Mean B-factor (mB**_f_**) for *Shaker* K**_V_** channel (*black*); KvAP (*purple*); CiVSP (*teal*); ehHv1 (*gray*), kHv1 (*green*); Vm-PomA (*pink*); Mpyr-TolQ (*brown*); Ppind-TolQ (*blue*); Osit-TolQ (*olive*); Pdb-TolQ (*cyan*); AqK-TolQ (*red*).

**Fig. S3.** Primary sequence for TolQ-like and PomA proteins for *Aquifex aeolicus* (a) and *Vibrio mimicus* (b). Bold letters indicate transmembrane segments for these proteins; shaded residues in gray show segments with similarity to S2, S3 and S4 segments in V-sensors. Key positions in V-sensor function are shown as circles, black circle indicates residues similar to Phe290 in *Shaker* channels, asterisk point to residues corresponding to the theoretical ancient *paddle*-motif, showing a high flexibility profile. Arrows indicate mutations where impairing swarming motility has been reported (*see* text).
